# To Live, Not Only Survive—An Ongoing Endeavor: Resilience of Adult Swedish Women Abused as Children

**DOI:** 10.3389/fpubh.2021.599921

**Published:** 2021-02-25

**Authors:** Hrafnhildur Gunnarsdóttir, Jesper Löve, Gunnel Hensing, Åsa Källström

**Affiliations:** ^1^School of Public Health and Community Medicine, Institute of Medicine, Sahlgrenska Academy, University of Gothenburg, Gothenburg, Sweden; ^2^Department of Health Sciences, University West, Trollhättan, Sweden; ^3^School of Law, Psychology, and Social Work, Örebro University, Örebro, Sweden

**Keywords:** resilience, well-being, childhood maltreatment, adult women, public health, qualitative

## Abstract

**Background:** A significant proportion of individuals exposed to maltreatment in childhood adapt positively in adulthood despite the adversities, i.e., show resilience. Little is known about resources and processes related to adulthood that promote resilience. Since women are overrepresented as victims of intrafamilial violence, understanding resilience among adult women is important.

**Objective:** To explore experiences of resilience among adult women who perceive well-being and well-functioning although being exposed to maltreatment during childhood.

**Participants and Setting:** This study included 22 women with experiences of childhood maltreatment, mean age of 48 years, living in Sweden.

**Methods:** Individual interviews were conducted and analyzed according to constructivist grounded theory.

**Results:** The process of resilience was experienced as an ongoing endeavor to live, not only survive, an internal process that interacted with external processes involving social relations and conditions. This endeavor was built on four interrelated resources: establishing and maintaining command of life; employing personal resources; surrounding oneself with valuable people; and reaching acceptance. These worked together, not in a linear or chronological order, but in up and down ways, turns and straight lines (now and then), through the process from maltreatment to well-being.

**Conclusion:** Resilience was found to rest on intrapersonal and interpersonal resources. Individual's inherent capabilities can be, depending on life circumstances and available resources, realized in a way that promote well-being and well-functioning despite severe adversities. Therefore, public health initiatives, social services, and policies should provide conditions that help women maltreated in childhood to live fully rather than merely to survive.

## Background

Childhood maltreatment, a major public health concern, violates children's rights to health, safety, and development and can negatively affect health and socialization that can last into adulthood (1). Therefore, society must establish and maintain effective initiatives that prevent child abuse and minimize the negative consequences of maltreatment. This strategy requires genuine understanding of processes and resources that promote resilience.

Between 1 and 16% of children in high-income countries are estimated to have been exposed to some kind of physical, sexual, or emotional maltreatment (1). A prior review suggests that in the Nordic countries, about 1% of children have experienced sexual abuse by a parent or a step parent, 3–9% have experienced physical abuse, and 7–12% have witnessed domestic violence (2, 3). Women are overrepresented as victims of all types of intrafamilial violence (1–4). Furthermore, women who experience poly-victimization (i.e., being victimized by more than one person such as parents and partners) tend to have more mental health problems during young adulthood (4). In addition, especially for women, being subjected to violence during childhood increases the risk of being subjected to violence later in life (5, 6). Well-known negative consequences of childhood maltreatment include criminality, mental health problems, and risky consumption of alcohol (1, 7). However, not all children who experience maltreatment experience negative consequences to the same extent. Some adapt positively despite severe adversities–i.e., they develop and display resilience behaviors (8–10). Previous research suggests that of those who experience childhood maltreatment, 15–47% develop appropriate social functioning and good mental health–i.e., 15–47% are assessed to experience resilience. This wide range of resilience rates is potentially explained by the use of different criteria for resilience (11). When studying what promotes resilience, previous research has mainly focused on resources related to childhood rather than adulthood (8, 12).

Originally considered mainly a personal trait, resilience is now considered a dynamic process that embraces individuals' interactions with their surroundings and manifests at different points in one's life (8, 10). The importance of increased understanding of the mechanisms and the resources needed to support resilience has been emphasized as a way to develop prevention practice and policy (9, 10). In childhood, emotional intelligence, problem-solving abilities, effective schooling, and positive relationship with competent and supportive adults have been identified as factors that contribute to resilience (8, 9). However, research regarding resilience resources for adults who experienced childhood maltreatment is scarce (12), but activities that enable victimized adults distance themselves from these adverse experiences and create positive opportunities that enhance their coping strategies seem to promote resilience (9). Furthermore, health-promoting behaviors and social resources in adulthood–e.g., appraisal, belonging, self-esteem, and tangible social support–might buffer negative effects of childhood adversities, including childhood maltreatment (13–15).

In summary, few studies have investigated resilience processes in adults. Given the higher prevalence of childhood abuse among girls, studies of women are especially important, also as more women than men experience mental illness. Because welfare practices and public health policies need a sound knowledge base, studies conducted in national context are needed. Thus, the aim of this study was to explore experiences of resilience among adult women in Sweden who perceive well-being and well-functioning although being exposed to maltreatment during childhood.

## Methods

### Study Design

This explorative qualitative study relies in constructivist grounded theory (16). Using purposive sampling, this study targeted women between 30 and 65 years old (i.e., working-aged women), an age when women are assumed to have had a reasonable chance to finish their education, build a family, or in some other way shape their adult life. The initial data analysis and data gathering were conducted simultaneously to allow for adding new themes to the interview guide if needed. Memos were also written after each interview, including the interviewers' summaries and preliminary codes. Constant comparisons were made throughout the process both between the interview notes of the two interviewers and between codes and categories in each step of the analysis. Data gathering was ended when the data were considered rich enough to compile a conceptual framework of the women's experiences of resilience.

### Participants

The women were recruited through an advertisement on Facebook with University of Gothenburg as a consigner (see @AWAREstudyGU on Facebook) that began with the following question: Have you been abused or neglected during your childhood? The advertisement was posted for 2 weeks in the Facebook news feed of female users between 30 and 65 years old and who lived in the middle and western regions of Sweden. Women were invited to participate if they were between 30 and 65 years old, had witnessed domestic violence or experienced any kind of physical, emotional, or sexual abuse and/or were neglected by a close adult during their childhood, and defined themselves as experiencing well-being and having a well-functioning life as an adult. The women responded to the advertisement by contacting one of the interviewers (first author or last author) by either e-mail or telephone. The women had the interpretative prerogative of their experiences of both the childhood maltreatment and the well-being/well-functioning when assessing who was to be included in the study. Two women opted out after the initial contact since they reappraised their situation as not experiencing well-being. The final participants included 22 women between 31 and 64 years old (mean age = 48 years). Of these 22 women, 13 described themselves as having been physically abused by their biological, step, or foster father as a child, and nine described having witnessed severe physical violence by their biological or stepfather against their mother. Nineteen described having been neglected by their primary guardian (most commonly their mother), and this was mainly emotional neglect. Nine had been psychologically/verbally abused by a parent and/or other household member. Five had been raped or sexually abused by their biological father and six by another household member or relative. Therefore, the vast majority described having been victimized in multiple ways as a child, repeatedly and during a substantial part of their childhood. No one described only occasional event of maltreatment.

### Data Collection

Data were collected during individual interviews between March and June 2018. All interviews were face-to-face in a place chosen by each woman, most often in a separate room at the University of Gothenburg or Örebro University, but some women chose to be interviewed in their home or at a café. The interviews were structured around three themes. In the first theme, the women were asked to describe how they defined and described their own well-being and well-functioning and why they chose to volunteer for the study. In the second theme, the women were asked to share their adversities in childhood. In the third theme, the women were asked to reflect on their life course and describe what experiences they perceived enabled their journey toward well-being and well-functioning. For example, they were asked to describe particular periods of life, events, persons, and activities that they perceived of as having specific importance. Follow-up questions were asked if needed, but primarily the women were encouraged to share their stories freely. The first and last author conducted 11 interviews each, lasting between 47 and 110 min. The interviews were audiotaped and transcribed verbatim.

### Analysis

The analysis was guided by the procedures of grounded theory as described by Charmaz (16). The analysis began during the data collection as the interviewers discussed the meaning of each interview, compared their understanding of the women's life stories and wrote memos immediately after the interviews. After all the interviews were completed, the transcriptions were read and systematically analyzed using the steps of initial, focused, and axial coding as well as constantly comparing the data [see Charmaz (16)]. The analyses were conducted in five steps. In the first step, the first author read and analyzed two interviews conducted by the last author and the last author read and analyzed two interviews conducted by the first author. During the reading the focus was on identifying processes and activities (initial coding) contributing to resilience according to the participants' life-stories from childhood to the day of the interview. To come to consensus regarding the meaning of the data in the first interviews, the processes and activities identified were compared, specifically focusing on their meaning and possible concepts that could capture the meaning. A picture of a pattern of the experiences of resilience started to emerge. In step two, the first and the last author read and analyzed two more interviews, one conducted by the first author and one conducted by the last author. Comparisons were made between the processes and activities identified by the both authors and consensus was reached about the understanding of processes and activities where the meaning was unclear. In addition, the processes and activities identified were compared to those coded in the two first interviews. During the second step, the codes were sorted into categories to capture different aspects of the resilience experienced (focused coding) in an attempt to clarify the pattern. Next, the procedure was repeated with two more interviews. In the third step, the first author read and analyzed the remaining interviews according to previous procedures and constantly compared this analysis to previously identified codes and categories as well as the emerging pattern. Specific attention was made to processes and activities potentially contrasting or challenging the categories previously constructed and the emerging pattern in order to be open to new aspects or dimensions. In the fourth step, the first and the last author together explored the properties of the codes and constructed subcategories reflecting different dimensions of the women's experiences of resilience (axial coding). In the fifth step, the first and the last author together with co-authors scrutinized the emerging results to make sense of all the processes and activities described. Eventually, the results were compiled into a conceptual framework of women's experiences of resilience explaining their well-being and well-functioning in adulthood despite abuse and neglect in childhood (theoretical coding). See Table 1 for example of the coding procedure.

**Table 1:** Example of the coding procedure.

**Quotation**	**Initial coding**	**Focused coding-category**	**Axial coding-subcategory**
*I am happy with myself, and it takes a lot for me to get angry or change that mood, but of course you have to work consciously with it and that, I think, starts with oneself and how one perceives things and chooses to perceive them. If you have that ability then, of course. So, I've probably always emphasized the positive things*.	I am happy with myself–I have probably always emphasized the positive things.	Employing personal resources	Embracing the joy of living
*And Mom could not cope. And I can see that; I didn't understand it back then. I was pretty angry with her for a while. However, I could see this later on; I understand that she could not cope. He knocked the shit out of her*.	I can see that; I didn't understand it back then. I understand that she could not cope.	Reaching acceptance	Finding explanations
*After all, I chose a man [...] a man who doesn't keep on rubbing up against me and doing other stuff. I chose a man who wouldn't keep going on with sexual abuse and stuff like that. I chose a man who is calm and caring and careful. Which may have its drawbacks in not being so adventurous [laughter]. But I made a choice there. A safe choice*.	I chose a man who is calm and caring and careful.	Establishing and maintaining command of life	Making strategic life choices

### Ethics

The study was approved by the regional board of ethical vetting in Gothenburg (dnr: 258-17). After being assigned to the study, the participants were provided written information via e-mail. The same written information was presented during the interview at which time they also could ask questions and, were informed about the possibility at any time to withdraw from the study. These issues were presented before they signed an informed consent document.

## Results

### To Live, Not Only Survive–An Ongoing Endeavor

During the analyses, an ongoing endeavor to live not just survive emerged as a core process of resilience. Surviving refers to basic needs and managing everyday duties of life. Living, on the other hand, refers to the right to experience joy and create emotional distance from negative childhood experiences by not letting those experiences define one's life or well-being. To live, not only survive incorporates the ability to function well and experience well-being despite childhood abuse, an attitude expressed in the following quotation: “Previously, I just was surviving, now I'm living” (Woman Q).

The core of the resilience processes associated with living not just surviving had to be constantly re-experienced–i.e., it was an ongoing endeavor. Therefore, the core category “to live, not only survive–an ongoing endeavor” captures resilience as a process without a defined beginning or an end: “It has been a fairly long process, so it is difficult to say an exact time, but I see it more as something that continues to evolve all the time” (Woman E).

**Figure 1:**
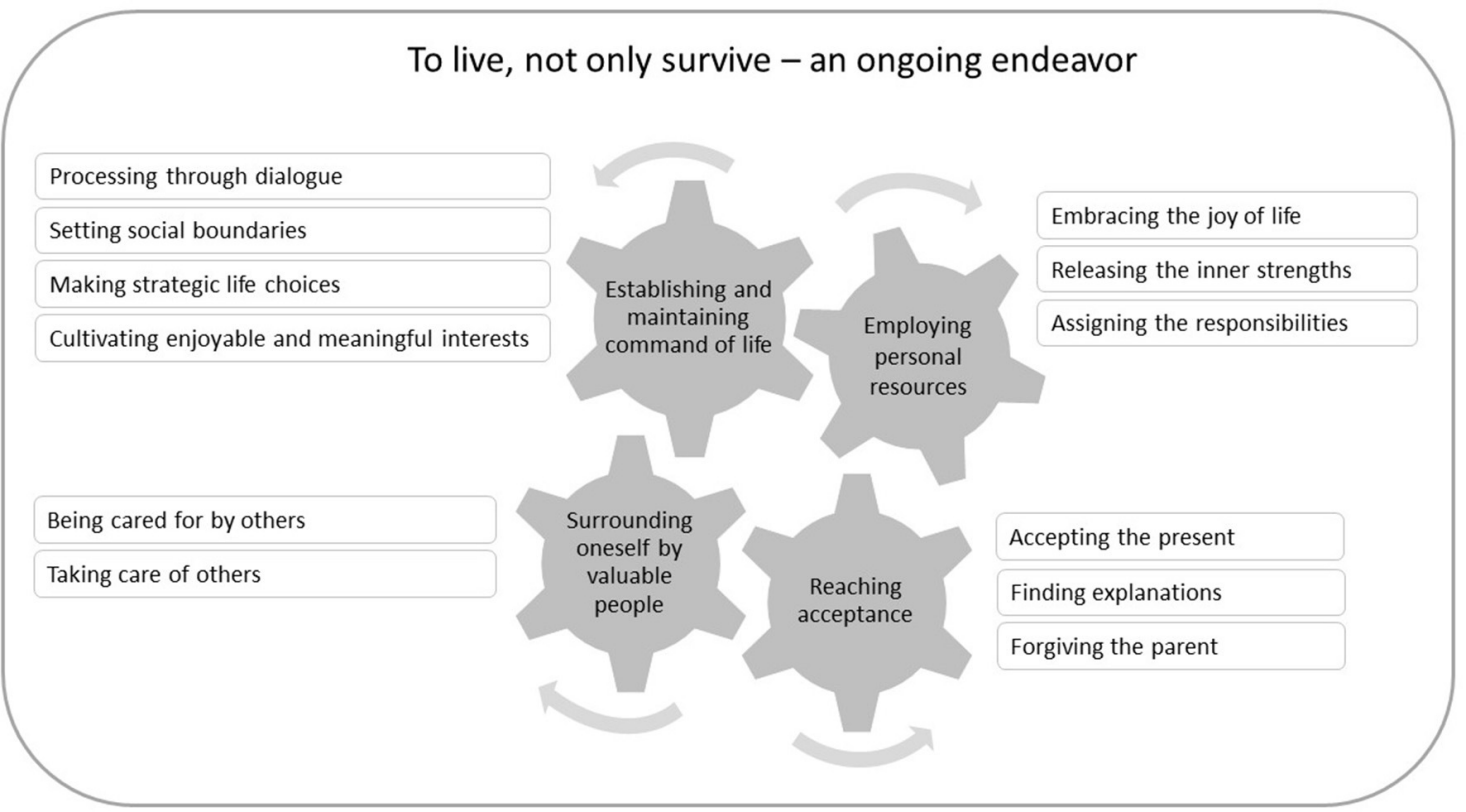
Conceptualization of women's experiences of processes of resilience in adulthood despite being exposed to maltreatment in childhood.

This endeavor was found to rest on four important types of resources. Not all women described having used all four types of resources, but all described having used more than one and it was clear that the resources typically enabled each other. Hence, we conceptualized the ongoing endeavor of living, not only surviving, as interrelated parts of a machinery with the different types of resources that drive the process of resilience (Figure 1). The resources enabling living are related to the women's intrapersonal efforts as well as interactions with others. These resources are described in the following categories: *establishing and maintaining command of life; employing personal resources; surrounding oneself with valuable people;* and *reaching acceptance*. These resources trigger or drive one another, but not in a specific order or as a chain of causality.

Although there are limits to how well the process of resilience can be conceptualized as machinery, we believe this metaphor illustrates that the resources can enable each other and that resilience is dynamic rather than static. For examples, *establishing and maintaining command of life* may demand *employing personal resources* and *surrounding oneself with valuable people* who activate a person's personal resources and ultimately *reach acceptance*. Allocating responsibility for the abuse to the abusing parent can help women accept the abusing parent's personal and environmental limitations. Furthermore, the intensity of the different resources varied over different periods of life, and the women described these resources to different extents. For some, the ongoing endeavor of living rather than just surviving began in childhood; for others, an additional trauma or other tough life experiences during adulthood triggered a turning point where they began their journey of ongoing living. For this latter group, after a period of experiencing a relatively smooth life even though primarily in the form of survival, something traumatic happened that urged these women to sort out their experiences and inner chaos:

*[I]t has been this process, that you [...] just like you do a puzzle. You find a piece there “ah, okay,” and then you find a piece there. And then more [...] that's what it has been like for me – that the more pieces I have found, the greater peace I have found within myself (Woman T)*.

The next section describes the types of resources and their dimensions in more detail. At the end of the description of each resource, the interrelations to the next resource described is considered.

### Establishing and Maintaining Command of Life

To establish and maintain command of life emerged as an important resource for the ongoing endeavor of living, not only surviving. The command of life could be established and maintained through processing experiences of abuse or neglect, managing feelings through dialogue with others or reflections within oneself, and setting social boundaries with others, mainly abusive parents. The command of life could also be established and maintained by making strategic life choices concerning education, work, living arrangements, family building, and partaking in enjoyable and meaningful interests.

#### Processing Through Dialogue

Processing through dialogue was important for establishing and maintaining control of life and comprised both internal dialogues and external dialogues with others. External dialogues included therapeutic conversations with professionals, informal conversations with friends, and sharing experiences with others with similar experiences. Being open and putting one's experiences and feelings into words was experienced as healing. The internal dialogue–i.e., self-reflection–was processed by writing letters or self-biographical notes or through meditation, all techniques used to confront one's feelings and to reach an understanding of the effects these experiences had on well-being and behavior:

*For me, it has been like, the more I talk about it, the more understanding and distance I get to what it is [...] to what it was like. It does not define me as a person today, but I carry it with me, I do, and always will (Woman T)*.

However, to put feelings into words, or “*to dare to meet oneself”* as one woman described it, required a different kind of effort and assistance. Some women did it mostly by themselves with minimal support from friends, relatives, or a professional. Others, however, tried a range of therapies before finding the one that suited them or met many professionals before finding someone they could trust. Whatever path they chose, the essence was to process their experiences actively, to do it with someone they trusted, and to distance themselves from the experiences of abuse even though they could not leave their experiences completely behind.

#### Setting Social Boundaries

The necessity of setting boundaries to focus on one's own needs and development or to protect oneself from abuse was highlighted. This necessity referred both to the relationship with the abusing parent[s] and to relations with others. Sometimes, setting boundaries included breaking contact with an abusing parent[s]. One woman described the strength she experienced by setting clear boundaries with her abusive father:

*I didn't want anything to do with [my] dad [...]. And I said to him, straight out and bluntly, “you can go to hell, I never want to see you again. You have ruined so much in my life.” And it made me feel so strong (Woman P)*.

At the same time, permanently breaking contact with parents was not described as a simple solution; it was described as challenging as emotional ties appeared to be strong. The woman cited above described how she later re-established contact with her father as she started to feel guilty over breaking contact. Another woman described the permanent break with her parents as a large hole in her life, but she believed she had no choice. She believed she needed to break from her parents so as not to be dragged down by their destructive behaviors. Although painful, the permanent break became her rescue. Setting boundaries also included clarifying distinctly for the parent that certain types of behaviors would not be accepted anymore, which especially was found needed when the women felt it necessary to protect their own children from their abusing parent[s]. One woman also described a fulfillment in being able to clearly set boundaries and speak her opinions or voice her needs, at times aggressively, to protect her (or even her friends') private sphere, a response to a threat she was unable to marshal as a child. Hence, control over one's life required setting boundaries in relation to others, establishing a few close friends instead of pressing oneself to interact with too many people, and, not the least, making all this clear to others.

#### Making Strategic Life Choices

Making strategic life choices embraced how women in young adulthood or in the transition to adulthood made choices and took actions to make the best out of their lives. One woman described how her choice of a husband was a strategic choice to ensure safety.

*After all, I chose a man [...] a man who doesn't keep on rubbing up against me and doing other stuff. I chose a man who wouldn't keep going on with sexual abuse and stuff like that. I chose a man who is calm and caring and careful. Which may have its drawbacks in not being so adventurous [laughter]. But I made a choice there. A safe choice (Woman J)*.

Making strategic life choices could also be about owning an apartment early in life, getting an education, and/or finding a job. It could also be about taking an opportunity to move on and try something new when an opportunity presented itself. Furthermore, making strategic life choices comprised choosing which relations were worth investing time and energy in. Overall, making strategic life choices required making choices to engage in things that contributed positively to one's life as well as opting out of things that did not benefit one's well-being.

#### Cultivating Enjoyable and Meaningful Interests

Women expressed that one part of controlling their life was to cultivate enjoyable and meaningful interests as engaging in activities and hobbies was described as contributing to well-being. These interests include cultural, physical, and cognitively-demanding activities such as solving a crossword puzzle, taking a course, and reading a book. In addition, taking care of animals was described as bringing delight and peace into one's life:

*And the horse makes demands. It gives a lot, but it also makes demands. You have to be present. You have to be calm. If you are not calm, the horse is not calm. So horses have been my salvation. And now they are my joy. And now I have my own horse (Woman U)*.

That is, the importance of not only engaging in activities but also in finding pleasure in small things in life was acknowledged.

The resilience resource of establishing and maintaining command of life enabled and forced the women to actively and strategically choose actions such as processing experiences, seeking help, setting boundaries, or filling life with meaningful activities. To choose such actions together with other resources contributed to the resilience process. In the next section, employing personal resources will be even more obvious and highlighted as an important resilience resource.

### Employing Personal Resources

An important part of the ongoing endeavor of not only surviving but also living was being able to use personal resources, innate or acquired, throughout the life course. Personal resources included the ability to embrace the joy of life, release one's the inner strengths, and allocate the responsibilities for experiences and situations in the past (the abuse) and the present (to live) to whom they belong.

#### Embracing the Joy of Living

Embracing the joy of living includes the importance of being able to feel joy and cheer even during the hardest of times. Some women described the ability to feel joy as an innate gift–i.e., they believed they were born happy. However, the ability to feel joy was also described as an active decision and skill acquired through life–i.e., an adapted approach. Embracing the joy of living could also be used strategically by rewarding oneself with enjoyable things when challenges had been managed:

*I am happy with myself and it takes a lot for me to get angry or change that mood, but of course you have to work consciously with it and that, I think, starts with oneself and how one perceives things and chooses to perceive them. If you have that ability then, of course. So, I've probably always emphasized the positive things (Woman B)*.

Therefore, taking advantage of the innate joy of living or making decisions to highlight the joyful things in life was considered an important contribution to well-being and well-functioning.

#### Releasing Inner Strength

The perception of an inner strength was also an important contributor or an explanation for why the women experienced well-being and well-functioning in everyday life in spite of their childhood adversities. The internal strength had both a dimension of an innate personal trait that had been of great help in childhood and a dimension of a quality they was adapted or was released because of the difficulty they were experiencing. One woman described this experience as her “lifeblood”: No matter how exposed or how broken she felt, her “lifeblood” always kept her up.

*I have given a name to this thing that I don't really know what it is. I call it my lifeblood, and it is completely unyielding. So, it doesn't matter how much of a difficult position I am in or how much I [...] no matter how hard life has been, somehow it has bounced back again (Woman F)*.

In general, however, the women found it difficult to explain their internal strength; that is, this internal strength was hard to put into words.

#### Assigning the Responsibilities

Assigning the responsibilities for past and present actions to where they belonged was an important personal resource. That is, these women embraced the conviction that they were not the cause of their parents' harmful acts nor deserving of the abusive treatment: their parents were the problem and they were innocent victims of the circumstances. To achieve well-being and a well-functioning life, it was important to clarify for oneself that the parents were responsible for the abusive treatment, but the responsibility to go on with life and convert the negative experiences to something useful was their own:

*It's all about, like, getting oneself out of this. And, that is not done by just sitting and feeling sorry for oneself. I also have a responsibility; I have to get a hold of my life (Woman R)*.

For some women, this had always been very clear; for others, it took them a long time to come to this insight. Assigning the responsibilities also included the present and the ability to move beyond victimhood, as pondering on victimhood was perceived to hinder well-being.

The resilience resource of employing personal resources could be enhanced by a significant person in the women's lives. At the same time, personal resources such as inner strength enabled them to establish valuable relationships, for example, with their children and to enjoy life even during hard times. The next section goes into detail about how other people can become resiliency resources.

### Surrounding Oneself With Valuable People

Surrounding oneself with valuable people was an important resource for being able to live and not only to survive and consisted of two dimensions: one describing the importance of opening up to being cared for by others and the one describing the importance of taking care of others. Being cared for by others embraced the importance of supportive relationships, both intimate relationships and relationships with professionals and others outside the women's immediate social circle, and the availability of others who can support the woman when needed. Taking care of others embraced the value of being responsible for the well-being of others, which made life worth living. Although the availability of valuable people, and particularly their care, was not described as completely up to the individual as surrounding oneself with valuable people demands being open to establishing such relationships and encouraging these relationships as well as finding people who would reciprocate these desires. These women had in one respect or another chosen to be surrounded with valuable people.

#### Being Cared for by Others

This dimension embraced the nurturing bonds the women managed to establish and maintain. A trustworthy relationship with a person they shared their life with, a husband or a partner, emerged central for the ongoing endeavor. A partner who was respectful and accepting, who the woman could rely on and build a new better life with, with or without children, was considered to contribute substantially to the well-being and well-functioning they experienced. Being cared for by others also embraced professional relationships, employers and/or colleagues, who recognized and acknowledged their abilities, confirming that they had value and a self-worth. These relationships also helped the women believe in themselves in a way they had not experienced. In addition, friend and sibling relationships were sources of support. The importance of being seen by someone outside their immediate social circle could be essential for the women's well-being and well-functioning–i.e., someone recognized the women's needs and/or their potential need for help or support, for example, a health professional or employer:

*She* [the child health care nurse] *kept calling me to meetings anyway; she wouldn't give up. And then, after a year she got me start talking [...]. So I mean, for me, it was a salvation that she understood. She was the only one who had seen and had understood (Woman A)*.

Being cared for by others also embraced how someone had taken action, for example, by not giving in even though the woman was not ready to talk or receive the help offered, but waited and tried again until she became ready or by putting a foot down and making the call, for example, to arrange someone professional to talk to.

#### Taking Care of Others

Taking care of others embraced the role children and family have played in the women's lives. Building one's own family could be a strategy to replace the family they never had as a child or a reason to live, strengthening their desire to keep struggling during hard times or when feeling life was meaningless. Although taking care of others could mean the women put their own feelings on hold, it could trigger the processing of adverse experiences from childhood.

*It has always been kind of my salvation, I would say, the kids and things. And in that way, I'm very grateful that I had a child early because I don't know if I'd have been here today if I hadn't had him to take care of (Woman E)*.

Becoming a mother also brought to light how wrong their parents had treated them during their childhood, and this could eventually bring strength and eagerness to never treat her children in same way.

Thus, being surrounded by valuable people who enabled the women to accept their experiences and life situations helped them process their experiences and feelings. In time, this also gave them strength to attempt to understand why they had been exposed to neglect. At the same time, acceptance of their situation and experiences could make them more receptive to the care of the valuable people with whom they were surrounded. The fourth aspect, the resilience resources encompassing acceptance, is further described in the next section.

### Reaching Acceptance

Acceptance could be reached through a better relationship with oneself (i.e., being a friend with oneself) and the life one was living. The process of acceptance was enabled by finding explanations for their childhood experiences and for some by forgiving their parents. Attempts to understand or find explanations to why their parents treated them the way they did was also a part of reaching acceptance, and some women even embraced reconciliation or forgiveness.

#### Consenting the Present

Consenting the present included accepting one's strengths and weaknesses and embracing the perception of being good enough no matter what. Consenting the present was described as the ongoing desire to battle one's problems, contributing to one's own well-being by living a life rather than just surviving. Similarly, being genuine about one's potential for chaos rather than presenting a calm façade was highlighted as important for attaining well-being:

*You have to allow yourself to be sad and think things are shitty, because sometimes it is. It is really shitty. But you have to remember to recall, even when it is at its worst, that it will get better again. It will be brighter again (Woman R)*.

Furthermore, consenting the present included embracing life as it is, with its ups and downs, and realizing that life naturally includes challenges that should be met with humility and serenity.

#### Finding Explanations

Finding explanations embraced the need to understand or explain why their parents were abusive. The explanations could be in the form of the parent having alcohol or psychological problems that triggered the abusive behavior or their parents' abusive behavior being the result of their parents' childhood abuse or neglect. Especially the father's abusive behavior was explained by illnesses or previous experiences being the cause, in part, of his lack of control. At the same time, it was found to be more difficult to explain why their mothers did not protect their children from their abusive fathers. This lack of maternal protection was explained by their mother being so totally broken down that she had to use all her energy just to survive. In the cases where the mother primarily was the abusing or neglecting one, the lack of protection from the father was more easily explained by his own vulnerability.

*And Mom could not cope. And I can see that; I didn't understand it back then. I was pretty angry with her for a while. However, I could see this later on; I understand that she could not cope. He knocked the shit out of her* (Woman U).

Finding explanations could lead to the conclusion that the parents did the best they could given the circumstances, a realization that was experienced as important for well-being.

#### Forgiving the Parent

The explanations and understandings of the parents' actions and situations could lead to forgiveness since, as one woman put it, “*you can love a parent at the same time you hate him or her”* (Woman 20). Receiving a request for forgiveness was perceived as a significant event and was found to be very relieving. Similarly, a parent's confession or insight about what he/she had done was wrong provided some relief:

*There came some kind of “sorry” in his own little way. And, for the first time, there, on his deathbed, I chose to actually sing for him of my own volition. It was big* (Woman P).

Thus, even a modest indication of regret that could be interpreted as the parent asking for forgiveness was described as very important. The resilience resource of reaching acceptance enabled the women to establish and maintain command of life at the same time enabled them to process their experiences through dialogue, ultimately facilitating their ability to reach acceptance.

## Discussion

This study adds to the research of resilience among adult women by exploring the process from childhood abuse and neglect to adulthood well-being and well-functioning. We found that this process was an ongoing endeavor to live, not only survive, an internal process that interacted with external processes involving social relations and conditions. This endeavor was built on four interrelated resources: establishing and maintaining command of life; employing personal resources; surrounding oneself with valuable people; and reaching acceptance. These four resources worked together, not in a linear or chronological order, but in up and down ways, turns and straight lines (now and then), through the process from abuse to well-being. Thus, this study found that resilience was something that continued throughout life with support of four important types of resources. Our findings regarding an ongoing endeavor agree with previous work conceptualizing resilience as a dynamic process (8, 9) and conclusions about resilience and recovery being an ongoing process (17, 18). Our findings contribute to earlier studies by providing more details that explain resilience as an interactive process consisting of four resources. The resources employing personal resources and reaching acceptance capture processes at an intrapersonal level that allowed the women to establish and maintain command of life and surround oneself with valuable people. The latter resources capture processes at an interpersonal level, activities that are done with others and depend on social conditions that also enable the internal processes. That is, these four resources work together and promote the ongoing endeavor for living, not only surviving. These results are in line with previous conceptualizations of resilience as a dynamic intrapersonal developmental process enhanced by interpersonal relationships and interaction with the environment (8, 9, 19). Our result that the ongoing endeavor is not linear agrees with Thomas and Hall (18), who identified three main patterns of trajectories when exploring recovery among women who experienced childhood sexual abuse. They identified a pattern of relatively steady upwards progression, a pattern of a lengthy roller coaster pattern with many ups and downs, and a pattern of struggle characterized by stagnation or downward progression. The steady upward progression and the roller coaster patterns illustrate a dynamic process of resilience and similar patterns could be glimpsed in the life stories of the women in our study. Our results extend Thomas and Hall's results by elucidating the ongoing endeavor to live a well-functioning life such that the trajectories move toward resilience and recovery rather than struggle and stagnation.

Previous research has concluded that turning points, including both positive and negative life-changing events, cause shifts and changes in directions of life trajectories that are central for developing resilience and the process of recovery (17, 18). Although our results did include occasional descriptions of additional trauma or other tough life experiences that in times could trigger the endeavor to live, not only survive, these did not emerge as sufficient for the process.

In our results, the rendering of resilience as an ongoing endeavor for living and not only surviving was found to rest on four resources. Some of the resources found in our study–e.g., releasing an inner strength, making strategic life choices, and being cared for by others–resemble previously described childhood resilience resources such as emotional intelligence, problem-solving personality, and positive relationship with competent and supportive adults (8, 9). The resources embracing the joy of life and releasing inner strength include being aware of one's own personal characteristics that can enable the resilience process, e.g., the perception of being born happy or perceived internal strength. These relate to personality traits, i.e., ego-resiliency, which previously have been described to play an important role in the process of resilience (8). Furthermore, our results include resources such as processing through dialogue, taking care of others, as well as being cared for by others that highlight the importance of social relations for the ongoing endeavor of living. These results agree with previous research that shows that safe and supporting relationships buffer the effects of maltreatment in childhood on various health outcomes among adult women (13, 15). Especially processing through dialogue accentuates the importance of having access to someone to talk to, someone with whom to process experiences. The women in our study described a variety of therapeutic contacts they used to process their experiences as well as informal contacts with friends, family members (e.g., partners or siblings), and colleagues. Thus, our results suggest that access to low threshold opportunities to diverse therapeutic methods may be a relevant public health initiative aimed at promoting resilience among women maltreated in childhood. Furthermore, in our results, the resources making strategic life choices and setting social boundaries reflect how the women used their internal resources in interaction with the environment (by moving away from the abusive context, getting an education, and/or finding a job) to find ways to distance themselves from their experiences and enhance their resilience. Thus, equal access to housing, employment, and education can be considered important for enabling the process of resilience. However, further research is needed to establish such evidence. Our results further add to the understanding of adult resilience resources by revealing the importance of pleasurable activities embedded in the endeavor of living, not only surviving, captured by the resources cultivating enjoyable and meaningful interests and embracing the joy of life. These results have not been prominent in previous research of adult resilience resources although some studies have described engaging in creativity and sports as useful resources for becoming less controlled by previous events (20).

The observed pattern of how the women explain their abuse differed for their fathers and their mothers, possibly reflecting gendered structures in society. Although the father or another male relative was the perpetrator in most cases, the women found it more challenging and complicated to explain why their mothers did not protect them from their abusive father than to explain their father's abusive behavior *per se*. These experiences might reflect the social construction of motherhood, including the expectation that mothers have the main responsibility for the care of the children. This perspective is particularly evident in the context of domestic violence where women's mothering has been framed as a determining factor in the protection of children and how the children are affected by the violence perpetrated by the father (21, 22). The lack of protection from the father in case of an abusive or neglecting mother was more easily explained by the father's own previous vulnerability. This view could be understood in the light of hegemonic masculinity where expectations of fathers are not mainly related to their engagement in the care of the children but rather in their position of dominance in the family (23).

### Strengths and Limitations

In our study, the women had the interpretative prerogative when assessing their childhood experiences, well-being and well-functioning, which can be considered a strength since it captures their own interpretations of resilience constructed in their own reality. However, it can also be considered a limitation since nuances in descriptions may have been lost and the chronological order of experiences may have been less precise, which can make the results even harder to transfer or compare to other women with experiences of childhood maltreatment. Nevertheless, memories of emotional events and traumatic experiences are suggested to be better recalled than more neutral events and experiences (24, 25). The initial purposive sampling (age, subjective well-being and well-functioning, and experience of childhood maltreatment) could be criticized for being too specified for grounded theory in that the defined borders already set the stage for the inductive theory. However, as stated by Breckenridge and Jones (26), researchers need some idea of where to start, and initial sampling can be a part of theoretical sampling before moving into simultaneous data collection and analysis based on constant comparison. The level of positive adaptation as criterion for resilience is a debated issue within the field: some researchers believe positive functioning in multiple domains of life is a requirement for resilience, whereas others believe positive functioning in only one domain is sufficient for resilience, depending on type of adversity (11). Furthermore, it has been argued that resilience should be assessed in terms of functioning that is relatively better compared to others with experiences of adversities at the same level (9) or based on other's views of how well the exposed individual is doing (27). However, resilience often becomes operationalized merely as the absence of psychopathology (11), which can be considered a narrow definition of positive adaption. Assessing resilient outcomes at different levels of life (e.g., intrapersonal, interpersonal, and/or societal) is another way of operationalizing resilience, allowing for wider understanding of positive adaption. Our study contributes to a wider understanding of positive adaption by exploring women's own interpretations of their well-being and well-functioning despite experiences of childhood maltreatment.

Several measures were taken to enhance the trustworthiness of the study, primarily through an accurate description of the performed procedures. To achieve credibility, we recruited participants with their own experiences of childhood maltreatment who were able and willing to share their stories, and we continued gathering data until we considered the data rich enough to provide a comprehensive answer for the study's focus. Confirmability was addressed by including several researchers in the data analysis (two in the initial steps and four in the last steps), opening up for alternative interpretations. In addition, quotes from the respondents were generously provided. The women included in this study were verbal, Swedish speakers, and active users of social media, who appreciated the opportunity to share their experiences as survivors rather than victims of childhood maltreatment. This should be kept in mind when the results are transferred or compared to other women with experiences of childhood maltreatment. To improve transferability, we provided information about the women's age, living context and type of childhood maltreatment they experienced up to an extent that would not risk revealing their identity.

### Implications

In prevention and health promotion within public health and social work, it is important to work from a knowledge base developed by systematic research. This qualitative study suggests that resilience is an ongoing process and that women use different types of resources to support this process. Several aspects of our results can inspire policy making and planning of health and social services. For example, available and affordable housing may be helpful for young women who need to set boundaries for themselves and flee abusive family situations. Furthermore, access to available and affordable counseling services may help women to process experiences of abuse and neglect. Furthermore, opportunities to cultivate meaningful interests and meet like-minded people are suggested as important resources for enabling women to distance themselves from their negative experiences and strengthen their social network, further developing their resilience. However, further quantitative research is needed to confirm the role of the identified resources in enhancing resilience among women maltreated in childhood.

### Future Directions

To advance the understanding of resilience among women exposed to childhood abuse or neglect, future studies should further explore how women perceive their well-being and well-functioning. To better understand gendered patterns, future studies should explore how men who experience childhood abuse or neglect develop resilience.

## Conclusions

The main result of this study–i.e., resilience is an ongoing endeavor to live, not only survive–supports the conceptualization of resilience as a dynamic process. Our results show that resilience among women who experience childhood abuse or neglect rests on intrapersonal and interpersonal resources. Furthermore, the results show that an individual's inherent capabilities, depending on life circumstances and available resources, can be vitalized/realized in a way promote well-being and well-functioning. Therefore, public health initiatives, social services, and policies should aim to create conditions with variety of resources available that help women who have experienced childhood abuse or neglect to live fully rather than merely to survive.

## Data Availability Statement

The dataset presented in this article are not readily available because the participants were guaranted that only the research group at University of Gothenburg and Örebro University would have access to the interview material. Requests to access the dataset should be directed to hildur.gunnarsdottir@gu.se.

## Ethics Statement

The study was reviewed and approved by Regional board of ethical vetting in Gothenburg (dnr: 258-17). The patients/participants provided their written informed consent to participate in this study.

## Author Contributions

HG and ÅK were responsible for the data gathering and the initial analysis. JL and GH contributed to the data analysis. HG drafted the main manuscript. All authors contributed with writing and intellectual feedback, contributed to the design of the study, and approved the submitted version.

## Conflict of Interest

The authors declare that the research was conducted in the absence of any commercial or financial relationships that could be construed as a potential conflict of interest. The handling editor declared a shared affiliation with one of the authors HG at time of review.
